# Elimination kinetics of ceftiofur hydrochloride in milk after an 8-day extended intramammary administration in healthy and infected cows

**DOI:** 10.1371/journal.pone.0187261

**Published:** 2017-11-02

**Authors:** Rongwei Han, Songli Li, Jun Wang, Zhongna Yu, Jiaqi Wang, Nan Zheng

**Affiliations:** 1 College of Food Science and Technology, Qingdao Agricultural University, Qingdao, P. R. China; 2 State Key Laboratory of Animal Nutrition, Institute of Animal Science, Chinese Academy of Agricultural Sciences, Beijing, P. R. China; 3 Haidu College, Qingdao Agricultural University, Laiyang, P. R. China; University of Illinois, UNITED STATES

## Abstract

Ceftiofur hydrochloride (CEF) is occasionally used for the intramammary (IMM) treatment of mastitis. This extralabel manner could result in a drug-residue violation of the milk. The objective of this study was to determine the elimination kinetics of IMM CEF in lactating dairy cattle. The pharmacokinetic profile of CEF after repeated IMM administration in nine healthy cows and nine *Staphylococcus aureus* infected cows was investigated, alongside determining the MICs of *Staph*. *aureus* field strains. The MIC 90 value for CEF in *Staph*. *aureus* field strains (n = 31) was 0.25 μg/mL. The t >MIC CEF values for low- production quarters were longer than those for high- and mid- production quarters. The results showed that ceftiofur was detected in milk up to 108 h after the last infusion in both healthy and infected cows. Cows with low milk production eliminate IMM drugs more slowly than cows with higher production. Our findings suggest that this extralabel use is not encouraged and a prudent use is recommended for mastitis therapy. The use of CEF should be reserved for infections where susceptibility tests indicate its efficacy and when alternatives are not available.

## Introduction

Bovine mastitis remains a serious challenge to the worldwide dairy industry due to the high incidence rate, for example, of approximately 17% in the United States and 33% in China [1,2]. Worldwidely mastitis incurres considerable economic losses ranging from €61 to €97 per cow per year on an average farm [3]. For bovine mastitis episodes caused by highly contagious staphylococci or streptococci, an intramammary infusion (IMM) of antimicrobial agents is usually used to treat clinical mastitis[4,5]. This treatment has been confirmed as having a good therapeutic effect as an antimicrobial agent, and it can attain and maintain an adequate and effective drug concentration at the site of infection in mammary tissue [6,7]. Ceftiofur is a broad-spectrum, third-generation cephalosporin antibiotic for veterinary use. Ceftiofur hydrochloride (CEF) is approved only for intramuscular injection and for subcutaneous injection to treat respiratory infections and necrobacillosis in cows, and when administered according to the instructions, it does not result in drug concentrations in milk greater than the tolerance limit set by the Federal Drug Administration (FDA) and the Chinese Ministry of Agriculture (MOA) of 0.1 μg/mL [8,9]. The use of 3rd and 4th generation cephalosporins has been revised in food producing species in Europe and USA and the advice of a prudent use has been reported to limit the spread of microbial resistance. The use of CEF should be reserved for infections where susceptibility tests indicate its efficacy and when alternatives are not available [10]. However, CEF is occasionally used by bovine cow practitioners for an extended period of time in an extralabel IMM manner to improve the treatment efficacy in clinical mastitis. Such treatment has been termed “extended therapy” and involves “changing route of administration and therapy use”. There are many reports of extended therapy with an IMM antimicrobial increasing the microbiological cure rate for mastitis episodes due to *Streptococcus uberis*, *Staphylococcus aureus* and *Corynebacterium bovis* [11–14]. The reason is that IMM and the extended therapy’s drug efficacy can be maximized by maintaining drug concentrations above the minimum inhibitory concentration (MIC) at the site of the mammary infection for as long as possible [15].

However, the extralabel use of this drug should be strongly discouraged since extralabel intramammary administration of drugs will lead risk of residues and serious economic consequences for the producer and veterinarian if extralabel drug use did not include a sufficiently extended withdrawal interval [16]. In China, milk must be withheld from sale following any treatment of cows until the antimicrobial concentration decreases below the allowable tolerance concentration. Violative residues will adversely affect human health and milk product quality if the drug withdrawl time is not long enough. Therefore, ceftiofur residue data is required to enable appropriate milk-withdrawal recommendations to be made practitioners when the drug is used as an IMM over an extended time.

Most of the previous research has mainly focused on the efficacy of CEF therapy for infected cows undergoing extralabel IMM treatment. An 8-d or 5-d extended IMM CEF treatment of moderate clinical mastitis in lactating dairy cows was found to be significantly better than the 2-d treatment or non-treatment in the controls [11,17,18]. Few studies have examined the elimination and duration of IMM CEF residues in milk. Eight healthy cows receiving 200 mg of IMM CEF (2 doses/d) had the highest drug concentrations of 450 ug/mL in their milk 4–6 h post-treatment [19]. One study on milk from five 300 mg IMM CEF (2 doses/d) treated healthy lactating dairy cows found that the milk should be discarded for a minimum of 7-d [20]. These studies all focused on healthy cows, and, to the best of the author’s knowledge, no reports have yet provided information about the elimination kinetics of IMM CEF in the milk of infected cows. However, comparison of elimination kinetics of CEF in healthy and infected cows is not clear. In addition, data about the CEF concentration in milk over time and its elimination rate in high- and low-yielding cows is worthy of more detailed study.

Therefore, accurate CEF kinetic elimination data are essential to undergo particular restrictions and the application of precautionary withdrawal times for the extralabel use of an unauthorized administration route (IMM), the increasing duration of therapy (8-d) and the unlicensed mastitis. The main purpose of this study was to evaluate preliminarily the characterization of the elimination kinetics of CEF and its metabolite—desfuroylceftiofur acetamide (DCA)—in milk and serum using an 8-d extended therapy program of daily IMM in healthy and infected lactating Holstein cows.

## Materials and methods

### Animal selection

The study was conducted at the Tsingtao Boyu Farm in the Shandong Province of China. This herd consisted of 680 milking cows of which 150 cows were tested for *Staph*. *aureus* and 48% were found to be infected. From among the animals tested, 18 lactating cows, with body weights (BWs) ranging from 620 to 760 kg (688±53 kg) who were in their first or second lactations were selected. All cows were provided with a total mixed ration (TMR) drug-free diet and water *ad libitum*. At the onset of the study, the animals with milk yield between 90 and 180 days in milk (DIM) (125±43 d) were used. The cows were milked twice daily and produced an average of 25 kg milk/d. All the experimental procedures with cows used in the present study were approved by the Animal Care Advisory Committee at the Institute of Animal Science, Chinese Academy of Agricultural Sciences.

### Preliminary bacteriology

The procedures for the bacteriological sampling and testing were carried out as reported in Cagnardi et al. (2010). A 10 μL milk sample from each quarter was plated onto blood agar (Oxoid, Basingstoke, UK) supplemented with 5% defibrinated sheep blood (Microbiol Diagnostici, Cagliari, Italy). Then the plates were incubated aerobically at 37°C for 24 h and suspected *Staph*. *aureus* was confirmed by the coagulase test and using the ID32Staph reference (Bio-Merieux, Marcy-l’Etoile, France).

The somatic cell count (SCC) was determined electronically (Fossomatic method; Foss Electric A/S, Hillerød, Denmark). Based on the bacteria and SCC test results of the milk, 18 cows were divided into a healthy (group H, *Staph*. *aureus* negative, SCC < 200,000 cells/mL) and an infected (group I, *Staph*. *aureus* positive, SCC > 200,000 cells/mL) group. Each group, consisted of three cows with low milk production (15–18.9 kg/d), three cows with mid milk production (23.2–27.6 kg/d) and three cows with high milk production (30.2–33.7 kg/d). Three quarters of 3 cows were discarded among healthy group due to high SCC, while 5 quarters of 5 cows were discarded among infected group due to *Staph*. *aureus* negative. Subsequently, 33 healthy quarters with high (n = 11), mid (n = 12) and low (n = 10) production and 31 infected quarters with high (n = 10), mid (n = 10) and low (n = 11) production were used for the experiments.

### MIC determination

Thirty-one isolates from infected quarters (*n* = 31) were tested for antimicrobial susceptibility to CEF by determining the MIC per the microdilution broth method, as recommended by the National Committee for Clinical Laboratory Standards (NCCLS, 2002). The CEF (EXCENEL RTU, Sterile Suspension, 50 mg/mL of ceftiofur as hydrochloride salt, Pharmacia Animal Health, Kalamazoo, Mich, United States) was dissolved and diluted in sterile distilled water. Isolates were prepared by diluting an overnight Mueller–Hinton broth culture in buffered saline solution to a density of 0.5 on the McFarland turbidity scale. For each isolate, the MIC was defined as the lowest concentration of CEF at which bacterial growth was completely inhibited. A reference strain of *Staph*. *aureus* (ATCC 29213; American Type Culture Collection, Manassas, VA) was inoculated as a control.

### Treatment and sampling

The two groups of cows were housed in different barns and were moved to a quiet room for milking. Milk samples were taken at 12h intervals (at 0600 and 1800 h) at milking time during treatments. Pre-milking procedures included dipping the teats with 0.5% iodine solution (Merck, Germany), allowing 30 s of contact time and wiping each teat dry with an individual paper towel. At milking time, a 5– mL foremilk sample was removed from each gland before attaching the cluster. Milking was performed using a homemade portable bucket milking machine, which could collect the milk from the 4 quarters to 4 milk pails, respectively. The milk from each gland of all the test animals was diverted from the bucket using the portable milking device. After the mammary glands and teats felt empty and no milk was seen entering the device for 30 s, the quarter milking devices were left in place. The milk production for each gland was measured with a 4000- mL graduated cylinder. A well-mixed 20- mL milk sample was collected from the quarter milking device at each milking and was frozen at –70°C until analysis.

After milking and teat disinfection at 0600 h every day, 125 mg IMM CEF (Excenel, Pharmacia Animal Health, Kalamazoo, Mich) was administered to each quarter of each cow every 24 h for eight consecutive treatments. Each cow reached a maximum dose of 500 mg of CEF every 24 h. After the last administration, sampling of milk was conducted at 12- h intervals for 5 consecutive days. Bacteriological cure was achieved if an infection was negative for the presence of *Staph*. *aureus*.

Blood samples were collected from one cow in each high–, mid–and low–milk production group separately. Subsequently, total 3 blood samples from healthy group and 3 blood samples from infected group were obtained. The jugular vein blood samples were drawn at t0 (before drug treatment) and after each drug administration at 2, 8, and 12 h. After the last administration, sampling of blood was conducted at 12- h intervals for 5 consecutive days. Subsequently, the samples were centrifuged (1,500 × *g*, 10 min at room temperature) to obtain the serum and were stored at –20°C pending the assay.

### Drug testing

High-performance liquid chromatography (HPLC) was used to analyze CEF and its metabolites by use of a previously described method [20]. First, all the ceftiofur and its metabolites from a 5–mL milk or serum sample were converted to desfuroylceftiofur acetamide (DCA) and then further purified using an Oasis hydrophilic-lipophilic-balanced (HLB) solid-phase extraction column. A 2–mL sample of the raw milk or serum was centrifuged to remove the fat, and 5 mL of 0.4% w/v dithioerythritol in borate buffer was added. The mixture was adjusted to pH 9.0 with NaOH solution and incubated in a water bath for 15 min at 50°C after vortex mixing for 5 min. Three milliliters of 14% w/v iodiacetamide in phosphate buffer (pH 7.0) was added and reacted for 30 min. Then the pH was adjusted to 2.5 with phosphoric acid and loaded onto a balanced Oasis HLB solid-phase extraction column. After washing with 5% methanol solution in water, the analytes were eluted with 2 mL of acetonitrile/methanol solution (20:80 by volume) and evaporated to dryness at 48°C under nitrogen. The mixture was then reconstituted with acetonitrile and water (15:85 by volume). After the solution was filtered through a 0.22-μm polyvinylidene difluoride membrane, 5 μL of the final extract solution was analyzed by HPLC with a C18 reversed-phase column at a UV absorbance of 263 nm. The analytes were separated with a mobile phase consisting of acetonitrile and sodium phosphate monobasic (NaH_2_PO_4_, pH 3.2, 20 mmol/L) buffer (15:85 by volume). The DCA limit of detection (LOD) and limit of quantitation (LOD) calculated for an S/N of 3 and 10 were 0.01 μg/L and 0.05 μg/L, respectively. Good linearity was found in the assay with the coefficient of determination (R^2^ = 0.9991) and the coefficient of variation was 5.7%. The CEF concentration in the milk was calculated by multiplying the [DCA] by the molecular weight ratio of CEF (523.9 g) to DCA (486.5 g).

### Elimination kinetic analysis

Elimination kinetic modeling after the last IMM infusion on d 8 was performed using calculated CEF concentrations for 5 d and 10 time points. The time required for the CEF in the milk to first decrease below the Chinese MOA and FDA tolerance concentration of 0.1 μg/mL after the last IMM infusion was determined.

As reported by Stockler et al. (2009) and Zonca et al. (2011), a noncompartmental model was fitted using an intravenous bolus model (WinNonlin model 201) and this was carried out on milk and serum drug concentrations to analyze the concentration–time profile and obtain comparable kinetic parameters. The mean residence time (MRT) was calculated using the standard equation MRT = AUMC/AUC, where AUMC is the area under the moment curve and AUC is the area under the milk or serum concentration–time curve [21].

### Data analysis

As drug quantification was carried out on samples from single quarters, results from group H were identified as healthy quarters (HQ = 33), whereas the results from group I were identified as infected quarters (IQ = 31).

Differences for milk and blood samples between healthy and infected cows and differences for milk samples among high, mid and low milk producers were investigated using the statistical analysis system (SAS) procedure (SAS Institute Inc., Cary, NC). An unpaired *t*-test with Welch correction (variances unequal) was performed on the elimination half-life, maximum concentration, MRT, and t > MIC. The ANOVA test was performed to evaluate differences among groups with different milk production rates. Data are presented as the mean ± standard deviation and P < 0.05 was considered significant in both tests.

## Results and discussion

The pharmacokinetic parameters of the milk and serum are shown in Table 1. The drug inhibitory concentrations toward *Staph*. *aureus* field strains (*n* = 31) ranged from 0.10 to 0.55 μg/mL and the calculated MIC90 was 0.25 μg/mL. The t > MIC values in milk were 58±27 h and 49±34 h in healthy and infected quarters, respectively (*P* > 0.05).

**Table 1:** Mean (±SD) milk and serum pharmacokinetic parameters after intramammary administration of ceftiofur hydrochloride in healthy (HQ) and infected quarters (IQ) in healthy and infected cows.

Parameter	Milk		Serum	
	Healthy quarter	Infected quarter	Healthy cow	Infected cow
	(*n* = 33)	(*n* = 31)	(*n* = 3)	(*n* = 3)
t_1/2λz_ (h)	37.6±4.12 ^a^	35.7±6.13 ^a^	29.2±5.73 ^b^	23.11±3.87 ^b^
T_max_ (h)	12	12	2	2
C_max_ (μg/mL)	41.7±11.58^a^	50.8±14.23^a^	0.08±0.023^b^	0.07±0.035^b^
AUC _last_ (h.μg/mL)	2483.15±471.24 ^a^	2871.77±625.54 ^a^	0.93±0.12 ^b^	1.24±0.25 ^b^
AUC _inf_ (h.μg/mL)	2579.45±594.22 ^a^	3017.36±890.03 ^a^	1.07±0.55 ^b^	1.28±0.27 ^b^
AUMClast (h.μg/mL)	12300.21±5763.18^a^	14319.86±6824.36^a^	12.95±4.76 ^b^	30.97±13.23 ^b^
MRT_last_ (h)	4.91±1.12 ^a^	2.88±1.91 ^a^	18.14±3.72^b^	26.53±4.58 ^b^
t > MIC (h)	58±27 ^a^	49±34 ^a^		

t _1/2λz_: elimination half-time, T _max:_ time to reach peak milk concentration, C _max_: peak milk concentration

AUC _last_ (h.μg/mL): area under milk concentration-time curve, AUC _inf_ (h.μg/mL): area under milk concentration-time curve from 0 to infinity, AUMC _last_ (h.μg/mL): area under the moment curve, MRT _last_: mean residence time, t > MIC: time during which drug concentrations exceeded the MIC.

^a, b^ Different letters indicate statistically significant differences in the same row (*P* < 0.01)

The drug amounts in the serum samples were low in healthy and infected cows with a maximum concentration (Cmax) of approximately 0.08 μg/mL in all selected cows (Table 1). No differences were observed in the parameters of serum pharmacokinetics between healthy and infected cows.

However, for the parameters of t_1/2λz_ and t>MIC, in all healthy quarters, there are significant differences between low- and mid-production quarters (*P* < 0.05), low- and high-production quarters (*P* < 0.05) (Table 2). Compared to high- and mid- production quarters, the elimination half-time and t > MIC values were longer in low production quarters. Similar results were also found for infected quarters. Thus, low production quarters will be more effectively treated and low-production mastitis-infected cows are easier to cure.

**Table 2:** Mean (±SD) milk pharmacokinetic parameters after intramammary administration of ceftiofur hydrochloride in healthy and infected quarters with high, mid and low milk production.

Parameter	Healthy quarter (*n* = 33)			Infected quarter (*n* = 31)		
	High production	Mid production	Low production	High production	Mid production	Low production
	(*n* = 11)	(*n* = 12)	(*n* = 10)	(*n* = 10)	(*n* = 10)	(*n* = 11)
t_1/2λz_ (h)	27.6±3.52^a^^b^	23.3±4.11^b^	41±0.71^c^	31.3±2.93^a^	26.5±5.38^a^^b^	39.61±1.42^c^
T _max_ (h)	12	12	12	12	12	12
C _max_ (μg/mL)	39.2±13.91^a^	35.2±9.05^a^	49.3±15.22^b^	49.4±11.35^b^	43.6±8.33^b^	60.2±17.48^c^
MRT _last_ (h)	3.36±0.57^b^	5.11±1.23^a^	6.26±1.07^a^	3.04±0.95^b^	2.45±0.73^b^	3.04±1.21^b^
t>MIC (h)	28±7^a^	38±5^a^	88±11^b^	34±6^a^	28±12^a^	85±13^b^

t _1/2λz_: elimination half-time, T _max:_ time to reach peak milk concentration, C _max_: peak milk concentration

MRT _last_: mean residence time, t>MIC: time during which drug concentrations exceeded the MIC.

^a, b, c^ Different letters indicate statistically significant differences in the same row (*P* < 0.01)

**Fig 1:**
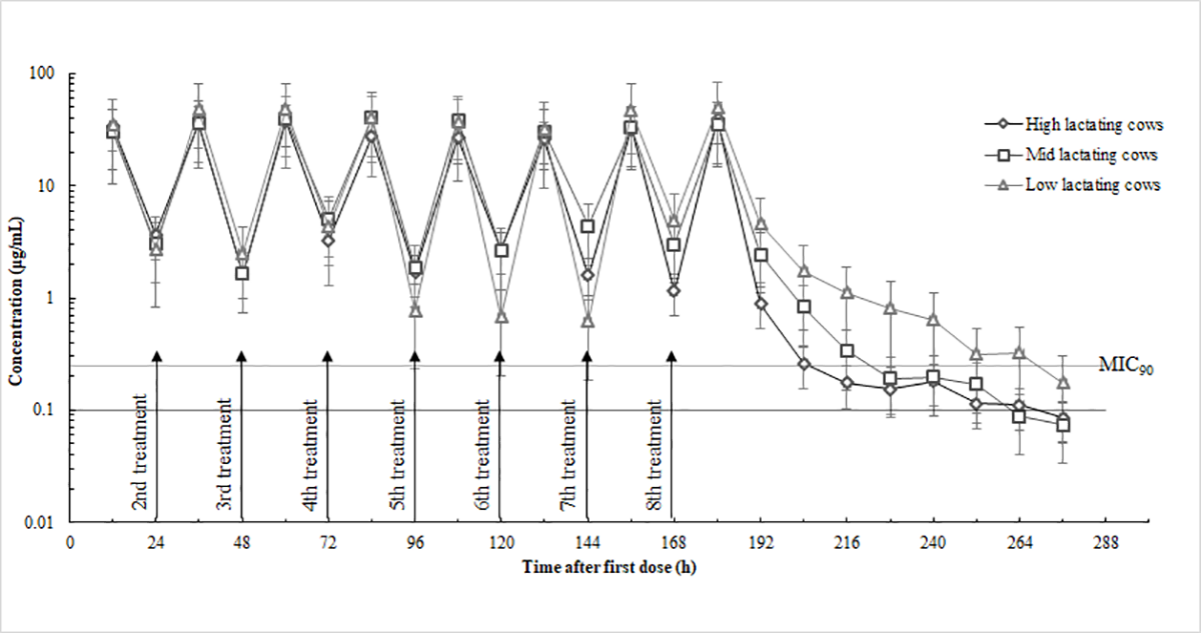
Milk concentration in nine healthy lactating cows' quarters with high (*n* = 11), mid (*n* = 12) and low (*n* = 10) production after eight infusions of ceftiofur hydrochloride at 24-h intervals, 125 mg/quarter, into all four quarters, plotted with MIC _90_ (0.25 μg/mL).

**Fig 2:**
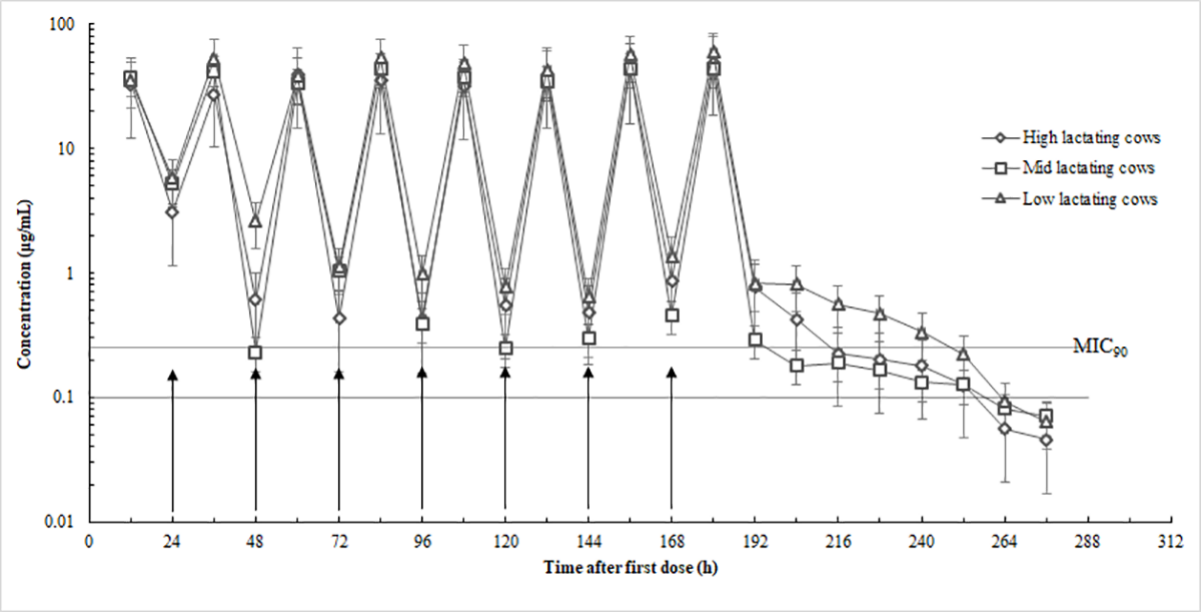
Milk concentration in nine infected lactating cows' quarters with high (*n* = 10), mid (*n* = 10) and low (*n* = 11) production after eight infusions of ceftiofur hydrochloride at 24-h intervals, 125 mg/quarter, into all four quarters, plotted with MIC _90_ (0.25 μg/mL).

**Fig 3:**
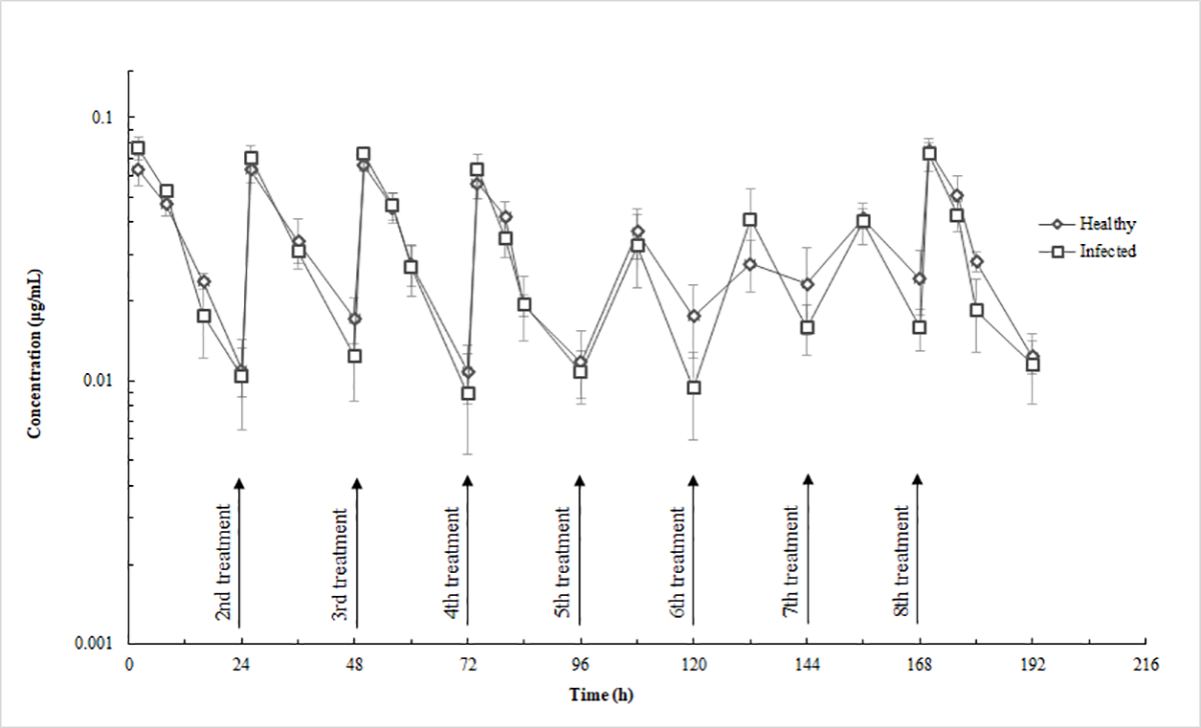
Mean-serum concentration (±SD) in healthy (*n* = 3) and infected (*n* = 3) cows administered eight intramammary infusions of ceftiofur hydrochloride at a dose of 125 mg/quarter each 24 h. Arrows indicate time of daily infusion of ceftiofur into all four quarters.

Ceftiofur was detected in milk up to 108 h (the ninth milking) after the last infusion in both healthy and infected cows (Figs 1 and 2). The mean concentration of CEF in the milk was below the Chinese tolerance concentration for saleable milk (0.1 μg/mL) at 108 h for healthy-cow quarters (high-, 0.08 ± 0.03 μg/mL; mid-, 0.07 ± 0.04 μg/mL) except in low production cows (0.18 μg/mL) and 96 h for infected-cow quarters (high-, 0.05 μg/mL; mid-, 0.07 μg/mL; low-, 0.06 μg/mL) after the last IMM infusion (Figs 1 and 2). Concentrations of CEF in the serum were far below those in the milk with the maximum value below 0.1 μg/mL (Fig 3). In the serum of selected animals, CEF was detectable for up to 24 h in healthy cows and in infected cows (Fig 3).

Pharmacokinetic studies are usually carried out on healthy cows. However, various factors in unhealthy cows could modify drug behavior [22]. Therefore, the IMM administration of CEF in healthy cows and infected cows was investigated in this study. No significant differences were observed in the drug elimination rate in milk or in serum between healthy and infected cow quarters (Table 1). However, as the results show in Figs 1 and 2, cow quarters with high- and mid-production rates eliminate the CEF more quickly after the last IMM treatment than cow quarters with low production do, both in healthy and infected cows. Earlier reports have suggested that drug elimination rates are influenced by the level of milk production [23,24,20,25].

The efficacy of β-lactam antimicrobial agents depends on the length of time the infectious agent is exposed to concentrations above the MIC [26]. Failure to maintain CEF concentrations above the MIC for a sufficient time in cow quarters with mastitis may result in treatment failure. As shown in Figs 1 and 2, the MIC90 values of CEF for *Staph*. *aureus* field strains were low (0.25 μg/mL) and the drug concentrations in the milk were maintained above the MIC90 for a long period. In both healthy and infected quarters, the elimination half-lives in milk were similar: 37.6±4.12 h and 35.7±6.13 h, respectively (*P* > 0.05), and the calculated t > MIC values were also comparable. The results indicated that in all quarters, the *Staph*. *aureus* field strains were exposed to drug activity for a long period and the CEF showed good efficacy against the pathogenic microorganism. As reported by Owen et al. (1999), the overall positive CEF performance after IMM injections against mastitis was also confirmed [19]. At the end of the study, milk from the infected quarters was monitored for health conditions and the degree of infection. The results were negative for *Staph*. *aureus*, thus confirming the efficacy of CEF. This was consistent with the cure efficacy in Oliver et al.’s (2004) study (125 mg CEF, IMM 8 times, 24-h intervals, 8 d).

In the current study, the CEF concentration in single-quarter milk when all four glands were treated with 125 mg of IMM CEF, eight times at 24-h intervals was below the FDA tolerance concentration of 0.1 μg/mL by 108 h in healthy quarters except in low production cows and by 96 h in infected quarters after the last infusion. This was not consistent with the findings (150mg CEF, IMM 2 times, 12-h intervals, discard time 7 d) of Smith et al. ‘s (2004) study. The reason may be due to the different IMM dosages and frequencies. Smith et al. ‘s (2004) study used a larger IMM dosage and quicker injection frequency. Figs 1 and 2 also indicate that the drug elimination rate was influenced by the level of milk production (Table 1). The results were consistent with previous studies [23,24,20,25]. Further investigations are needed to determine the reasons as to why milk production affect the pharmacokinetics and treatment efficacy of extended IMM CEF therapy by increasing the number of animals tested.

## Conclusion

The elimination kinetics of CEF product administered every 24 h via IMM in every quarter at a dose of 125 mg for cow mastitis therapy use was demonstrated in this work. The CEF MIC90 values for *Staph*. *aureus* field strains were low (0.25 μg/mL). There were no significant differences about the serum pharmacokinetic parameters as well as milk pharmacokinetic parameters between healthy and infected quarters. The elimination half-time and t > MIC values for CEF in low production quarters were longer than those in the high- and mid-production quarters. The results of this study revealed that prudent use of this extralabel manner of CEF was not encouraged for practitioners due to the risk of drug residues. The use of 3rd and 4th generation cephalosporins has been revised in food producing species in Europe and USA and the advice of a prudent use has been reported to limit the spread of microbial resistance. The findings from this study was a preliminary evaluation in elimination kinetics of CEF in extralabel manner use. Further studies are still needed to avoid the risk of drug residues entering the human food chain by determing a precautionary withholding time.
